# The Improvement of Left Atrial Function after Twelve Weeks of Supervised Concurrent Training in Patients with Heart Failure with Mid-Range Ejection Fraction: A Pilot Study

**DOI:** 10.3390/jcdd10070276

**Published:** 2023-06-29

**Authors:** Giuseppe Caminiti, Marco Alfonso Perrone, Valentino D’Antoni, Giuseppe Marazzi, Alessandro Gismondi, Sara Vadalà, Deborah Di Biasio, Vincenzo Manzi, Ferdinando Iellamo, Maurizio Volterrani

**Affiliations:** 1 Department of Human Science and Promotion of Quality of Life, San Raffaele Open University, 00163 Rome, Italy; 2 Cardiology Rehabilitation Unit, IRCCS San Raffaele, 00163 Rome, Italy; valentino.dantoni@sanraffaele.it (V.D.); giuseppe.marazzi@sanraffaele.it (G.M.); vada.sara21@gmail.com (S.V.); debbydibiasio20@gmail.com (D.D.B.); 3 Division of Cardiology and Sports Medicine, Department of Clinical Sciences and Translational Medicine, University of Rome Tor Vergata, 00133 Rome, Italy; marco.perrone@uniroma2.it (M.A.P.); gismondi.alessandro@outlook.it (A.G.); iellamo@uniroma2.it (F.I.); 4 Department of Humanities, Università Telematica Pegaso, 80132 Naples, Italy; vincenzo.manzi@unipegaso.it

**Keywords:** concurrent training, heart failure, left atrial function

## Abstract

Left atrial dysfunction is associated with exercise intolerance and poor prognosis in heart failure (HF). The effects of exercise training on atrial function in patients with HF with mid-range ejection fraction (HFmrEF) are unknown. The purpose of the present study was to assess the effects of a supervised concurrent training (SCT) program, lasting 12 weeks, on left atrial function of patients with HFmrEF. The study included 70 stable patients, who were randomly assigned into two groups: SCT with (three sessions/week) or a control (CON) group directed to follow contemporary exercise preventive guidelines at home. Before starting the training program and at 12 weeks, all patients performed an ergometric test, a 6 min walk test, and echocardiography. Between-group comparisons were made by analysis of variance (ANOVA). At 12 weeks, the duration of the ergometric test and distance walked at 6 min walk test presented a significant greater increase in SCT compared to the control (between-group *p* 0.0001 and *p* 0.004 respectively). Peak atrial longitudinal strain and conduit strain presented an increase of 29% and 34%, respectively, in the SCT, and were unchanged in CON (between-group *p* 0.008 and *p* 0.001, respectively). Peak atrial contraction strain increased by 21% in SCT, with no changes in CON (between-group *p* 0.002). Left ventricular global longitudinal strain increased significantly in SCT compared to control (between-groups *p* 0.03). In conclusions, SCT improved left atrial and left ventricular function in HFmrEF. Further studies are needed in order to verify whether these favourable effects of SCT on LA function are sustained and whether they will translate into clinical benefits for patients with HFmrEF.

## 1. Introduction

Heart failure (HF) is a progressive cardiovascular disease with significant morbidity and mortality that affects an increasing amount of people worldwide [1]. Traditionally, left ventricular (LV) remodelling, leading to systolic and/or diastolic dysfunction, has been identified as the most important determinant of pathophysiological derangements and clinical manifestations that characterize the disease [2,3]. These detrimental changes in the LV are also reflected at the atrial level. During HF, the left atrium (LA) is characterized by an adverse remodelling process, triggered by pressure or volume overload, ultimately resulting in LA dilation and atrial tachyarrhythmia [4]. Several studies have pointed out that LA remodelling plays a key role in determining the clinical burden of HF [5]. The development of LA enlargement has been associated with increased risk of incident atrial fibrillation, poor exercise tolerance, increased morbidity, and mortality in HF [6,7,8,9,10,11,12]. Changes in LA size are preceded by the onset of functional abnormalities [13]. LA dysfunction can be detected through two-dimensional speckle tracking echocardiography, which studies the deformation of atrial walls during the cardiac cycle. This technique permits one to distinguish three atrial phases: the reservoir strain, a phase of LA expansion that occurs during left ventricular (LV) systole; and the conduit and contraction strain, which correspond, respectively, to the early and late phases of LV diastole [14]. A reduced LA reservoir strain has been identified as an early marker of LV diastolic dysfunction and of raising LV filling pressure, and it is considered as the first step of the LA functional remodelling process occurring during HF [15,16]. Assessing LA dysfunction seems to provide more prognostic information than LA size itself in HF patients [7]. In particular, LA reservoir function, expressed as peak atrial longitudinal strain (PALS), has proved to be a powerful prognostic marker in HF independently from the left ventricular ejection fraction (LVEF) values [8]. In consequence of its growing clinical relevance, LA dysfunction is becoming a potential therapeutic target in HF. While improvements of LA function have recently been observed after some pharmacological therapies [17], it is not known whether exercise training can have any favourable effect on LA function. In this study, we focused on patients with HF with mid-range ejection fraction (HFmrEF), since this category of HF patients were underrepresented in previous studies involving exercise training. Regarding the exercise modality, in this study, we used a concurrent aerobic-plus-resistance training (SCT) program. This is the exercise modality more often adopted at our centre in patients with HF, since it has been proved to be superior to continuous aerobic training in improving functional capacity and muscle strength in these patients [18,19]. SCT has also shown favourable effects on LV diastolic function and has improved haemodynamics in HF [20]. However, there are no data about SCT effects on LA function indices. The purpose of the present study was to assess the effects of a 12-week SCT program on LA function in patients with HFmrEF and underlying coronary heart disease (CHD). We hypothesized that SCT would improve PALS and other indices of LA function in these patients.

## 2. Materials and Methods

### 2.1. Population

This study enrolled a total of 70 consecutive subjects, 57 males and 13 females, in NYHA class I/II, with a previous diagnosis of HFmrEF secondary to CHD. They were all patients referred to the outpatient clinic of the rehabilitation centre of San Raffaele IRCCS in Rome. Inclusion criteria were the following: stable clinical conditions (no hospitalizations for HF or acute coronary syndrome in the past six months and/or no changes on pharmacological therapy in the past three months); age over 45 years; LV ejection fraction ranging between 40–49%; stable sinus rhythm; normal or mildly enlarged LA volume (<41 mL/m^2^). Exclusion criteria adopted in this study were the following: baseline blood pressure levels at rest over 160/100 mmHg; severe heart valve diseases; hypertrophic cardiomyopathy; ergometric test showing signs and/or symptoms of myocardial ischaemia; uncontrolled arrhythmias; neurological and/or orthopaedic conditions contraindicating or limiting exercises; advanced chronic obstructive pulmonary disease with FEV1% <50%; symptomatic peripheral arterial disease. Patients were also excluded if they needed to change their pharmacological treatment during the study period.

### 2.2. Study Design

**Figure 1:**
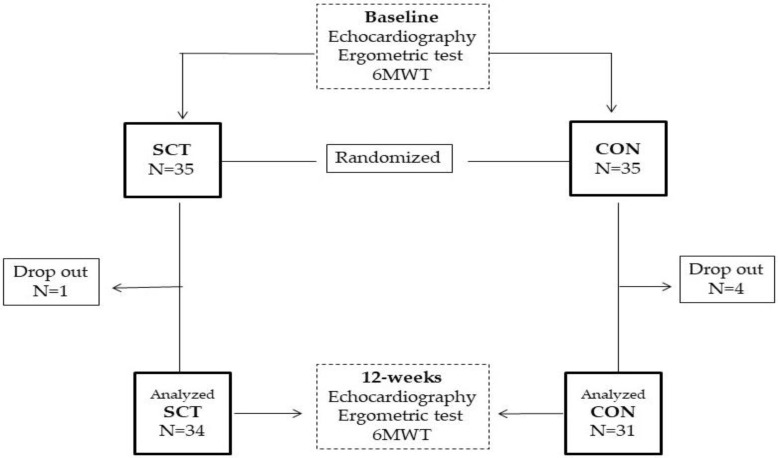
Study flow chart.

The study design is summarized in Figure 1. It was conducted as a prospective, longitudinal, randomized pilot study. Patients were randomly assigned on 1:1 basis to either a supervised combined training group (SCT) or a control (CON) group, performing unsupervised home-based exercises. The randomization code was developed with a computer random number generator to select random permuted blocks. The study complied with the Declaration of Helsinki and was approved by the local Ethics Committee of San Raffaele IRCCS (protocol number 33/2021). All patients gave written informed consent before entering the study. At baseline, all patients underwent a full clinical evaluation, which was divided into two days. The first day included collecting patient’s medical history; measuring body mass index (BMI), waist circumference, resting heart rate and blood pressure; performing a transthoracic echocardiography. The second day included performing 6 min walk test (6MWT) and ergometric test; assessing the 1-repetition maximum (1RM). The 6MWT was performed according to standardized procedures [21] and was supervised by a physical therapist. The ergometric test was performed on a treadmill (Mortara Instr, Bologna, Italy), and a standard Bruce protocol was adopted in each patient. At each stage of the test, the rate of perceived exertion (RPE) was assessed through the Borg 6–20 scale [22]. Before starting the assessment of 1RM, patients were allowed to familiarize, for at least 30 min, with dynamometers, and were supervised by a physiotherapist who had the task of teaching them how to perform properly the different exercises. The 1RM for each resistance exercise was assessed according to the following procedure: patients performed a warm-up set (8–10 repetitions) at 50–60% of their perceived 1RM; then, they were asked to perform one repetition at their maximal effort. The latter was repeated three times, with 2 min of rest between efforts, and the highest value of strength recorded was used as 1RM [23]. At 12 weeks, echocardiography, 6MWT, and ergometric tests were repeated, within a week from the last exercise session.

### 2.3. Echocardiography Assessment

Transthoracic echocardiography was performed in the supine position; a cardiovascular ultrasound Vivid E95^®^ (GE Healthcare, Chicago, IL, USA) with a 4.0-MHz transducer was used. All echocardiography examinations were performed by one experienced sonographer who was blinded to the type of patient’s allocation. During echocardiography, one-lead electrocardiography monitoring was conducted. All the echocardiographic images were stored digitally and reviewed offline; during that phase, an experienced technician performed deformation measures using a proprietary software (version 10.8, EchoPAC; GE Vingmed Ultrasound, Horten, Norway). Left ventricular end-diastolic volume (LVEDV) and left ventricular end-systolic volume (LVESV) were calculated from the apical two- and four-chamber windows; LVEF was assessed by the modified Simpson’s method. LA volume was measured from standard apical four-chamber and three-chamber views before mitral valve opening, at end-systole; Simpson’s biplane method of disks was adopted. The calculation of LA volume index (LAVI) was made by dividing LA volume by the body surface area of subjects. E/A ratio represented the ratio of peak left ventricle filling velocity in early diastole (E wave) to peak velocity flow in late diastole, during atrial contraction (A wave). LV E/e’ ratio was calculated as the ratio between E wave velocity and mean lateral and septal LV e’ wave velocities. Colour tissue Doppler tracings were obtained, with the range gate placed at the lateral mitral annular segments in the four-chamber view. LV global longitudinal strain (GLS) was measured through two-, three-, and four-chamber views. The detection of the LV endocardial boundary was automatically provided by the software; however, each time it was deemed appropriate, it was edited to conform to the visualized LV boundaries. The maximum negative value of strain during systole, as measured by the software, represented the maximum contractility for each segment. The average of these values from each segment was used to determine LV GLS. Measures of LA deformation tracking were carried out using the R wave as a starting point (R-R gating). Endocardial and epicardial contours of the LA were traced in the end-diastolic phase of the long-axis two-chamber and four-chamber cine images. The automatic contour tracking algorithm was used and manual adjustments were applied, if necessary. This algorithm places a set of control points on the middle curve of the myocardial wall in the reference phase based on the drawn endocardial and epicardial contours. The software program generated the longitudinal strain curves for each segment and a mean curve of all segments. Longitudinal strain measurements were subdivided into LA reservoir strain, conduit strain, and contractile strain [14]. Peak atrial longitudinal strain (PALS) was measured at the end of the reservoir phase (positive peak during LV systole); peak atrial contraction strain (PACS) was measured just before the start of the active contractile phase (positive peak during early diastole). Conduit strain was measured as difference of the strain value at the onset of atrial contraction minus mitral valve opening [24]. All strain values were taken by a single cardiac cycle. The intra-observer reproducibility of echocardiography measurements was assessed through the intraclass correlation coefficient (ICC) on 15 healthy voluntaries. Two echocardiography assessments were performed at different times (with at least a week between assessments). The ICC for all LA and LV parameters ranged between 0.87 and 0.98.

### 2.4. Exercise Training Programs

Patients of the SCT group performed the training sessions individually in the rehabilitation centre of our hospital. Patients’ heart rhythm was monitored by telemetry in the first session. The duration of the sessions was 60 min, and each session was preceded by 10 min of warm-up and concluded with 10 min of cool-down. The frequency of the sessions was set to three times per week. The training sessions were structured as follows: 40 min of aerobic exercise on the treadmill (Technogym, Cesena, Italy) with an intensity target of 13–14 RPE on the Borg scale (patients were allowed to change the treadmill settings in order to maintain the effort across the rehabilitation protocol); 20 min of resistance training involving the following exercises in this specific order: horizontal leg press, shoulder press, lat pulldown, leg extension, chest press, and seated row; 2 sets of 10 repetitions at 60% of the 1RM performed for all the exercises, with a 2 min rest period between sets. The 1RM strength tests were repeated every month in order to update the percentages of the exercises for training sessions. The exercise sessions were supervised by a rehabilitation team, including a sport medicine physician with experience in exercise prescription, a nurse specialized in the cardiac rehabilitation, and a physiotherapist, who helped the patient set up the treadmills for the aerobic component of the training and checked the executions of the resistance training exercises. No motivational strategies were adopted during the rehabilitation sessions of the SCT group. The adherence to the training program was calculated as follows: (attended sessions/planned sessions) ×100. The CON group was advised to follow contemporary preventive guidelines [25], and at the beginning of the study, a short training manual and educational materials were delivered to them. CON patients did not receive any supervision nor wearable device for physical activity tracking throughout the study. Moreover, no contact was established with these patients during the study period. Patients of the CON group were summoned for the final evaluation at 12 weeks, and on that occasion, they were asked about the number of exercise sessions performed at home.

### 2.5. Statistical Analysis

We did not find previous studies investigating the effects of exercise training on LA function in HFmrEF patients; therefore, we conceived this research as a pilot study. The sample size was then chosen according to the expected number of patients referred to our centre during the recruitment period and to available resources. We estimated that about 40% of them would have agreed to participate to the study. Data are presented as mean ± standard deviation (SD) or percentage, where appropriate. The assumption of normality was checked using the Shapiro–Wilk hypothesis test. Pre- and postexercise data were assessed using a repeated-measures two-way ANOVA, with Bonferroni corrections for post hoc testing. The Pearson correlation coefficient test was used to measure the strength of a linear association between two variables. The level of significance was set at *p* < 0.05. All analyses were performed using a commercially available statistical package (SPSS for Windows 20.0, Chicago, IL, USA).

## 3. Results

### 3.1. Baseline Characteristics of Patients

Patients of the SCT and CON groups had similar baseline characteristics, including the number of drugs used for treating CAD and heart failure (Table 1). The mean age of the whole sample was 68 years. Most patients of both groups had a previous myocardial infarction (SCT = 80%; CON = 74%); 76% of SCT and 79% of CON were overweight or had systemic arterial hypertension. Most patients were taking betablockers and angiotensin-converting enzyme inhibitors or angiotensin receptor blockers. A total of 5 patients of the 70 initially screened dropped out of the study. Overall, three patients (one in the SCT and three in the CON group) dropped out before completion, for their unwillingness to continue the study. Also, one patient of the CON group was excluded from the analyses due to changes in pharmacological therapy. Therefore, 65 out of 70 patients completed the study, and their data were evaluated in the analysis. The average number of sessions performed by patients of the SCT group was 34.6 ± 4.2, and the adherence rate was 94.4%. Patients of the CON group declared an average number of sessions of 13.9 ± 6.5. Moreover, nine patients of the CON group declared that they did not exercise during the study period. No adverse events occurred during the study period in the two groups.

### 3.2. Between-Group Comparisons

**Figure 2:**
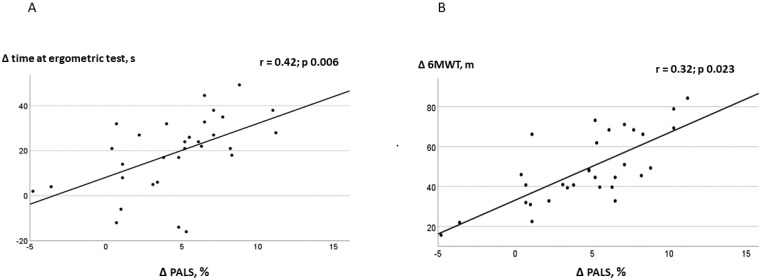
Correlations between PALS changes (12 weeks versus baseline) and changes in time at ergometric test (**A**) and distance walked at 6MWT (**B**).

At the end of the study, the duration of the ergometric test increased significantly in the SCT group compared to CON (SCT from 325.2  ±  46.1 s to 415.3  ±  51.7 s, *p* 0.001; CON from 332.5  ±  54.3 s to 348.3  ±  79 s, between-group *p* 0.0001); 6MWT also increased significantly in the SCT group compared to CON (SCT from 429.6  ± 52.8 m to 512.4  ±  66.3 m; CON from 422.2  ±  47.3 m to 468.3  ± 62.4 m, between-group *p* 0.004). At the end of the study, PALS and conduit strain increased significantly in the SCT group compared to CON (between-group *p* 0.008 and 0.001, respectively). PACS increased in the SCT group compared to CON (between-group *p* 0.002; Table 2). E/e’ was unchanged in the SCT and CON groups. GLS was increased in the SCT compared to CON (between-group *p* 0.03). In the entire population, baseline values of PALS were not significantly correlated to the baseline duration of the ergometric test (r = 0.24, *p* 0.092) and baseline distance walked at 6MWT (r = 0.12, *p* 0.154). The change in PALS in the SCT group was significantly related to changes in the duration of the ergometric test (r = 0.42, *p* 0.006) and to changes in distance walked at 6MWT (r = 0.32, *p* 0.023; Figure 2). There were no significant correlations between PACS and duration at exercise training and distance walked at 6MWT. Atrial volume, E/e’ ratio and E/A did not change at 12-weeks compared to baseline in either group.

## 4. Discussion

### 4.1. Main Results and Clinical Context

Left atrial dysfunction, assessed by speckle tracking echocardiography, is considered the first step of the atrial remodelling process that occurs during HF. A reduced LA reservoir strain, the most studied LA functional parameter, proved to be an independent predictor of reduced exercise tolerance and poor prognosis in these patients [6,12]. Developing specific treatment strategies aimed at improving LA function could have a positive impact on the course of the disease and on the symptoms of the patients [26]. In this pilot study, we investigated the effects produced by a supervised concurrent exercise program, lasting 12 weeks, on LA and LV function parameters in HFmrEF patients. We observed that after 12 weeks, resting values of PALS and PACS as well as LV GLS were significantly increased in the SCT group compared to baseline values and to control. To our knowledge, no previous research has addressed this topic in HF. Studies assessing the effects of exercise training on LA function are sparse, having been mainly conducted in athletes or in healthy subjects, and have shown conflicting results [27,28,29]. Despite being very preliminary, data of the present study constitute the first demonstration that a nonpharmacological intervention could be effective in improving indices of LA function in HFmrEF. A similar result was recently obtained, in HF with reduced EF, by different research groups using the same pharmacological approach [17,30,31,32]. In these studies, the administration of sacubitril-valsartan reduced LA size and improved LA reservoir strain and LV GLS, with these benefits typically observed after 6 months of treatment. It is noteworthy that those previous pharmacological studies did not consider the effect of exercise training and enrolled patients with HF in a more advanced stage compared to our study. For example, in the research by Correale et al. [32], the average LVEF was 27%, and half of the study population was in NYHA class III. Moreover, none of the patients enrolled in the present study was taking sacubitril-valsartan, since this drug is usually prescribed to patients with EF lower than 40%. The design of the present study does not allow us to investigate mechanisms underlying the observed benefits of SCT in HFmrEF. However, the parallel improvement of PALS and PACS lets us assume that SCT induces a better emptying of the LA, probably as a consequence of an improved LV diastolic function. In accordance with this hypothesis, there are two observations: PALS has been proven to be a more reliable marker of LV diastolic dysfunction then E/e’ ratio [33]. Moreover, positive effects of SCT on LV diastolic dysfunction have been previously described [34]. The improved LA function may have contributed to a restoration of the Frank–Starling mechanism at the LV level, and this could explain the increase in LV GLS that we observed. However, this hypothesis should be taken with caution, because not all data that we obtained in this study agree with this interpretation; for example, we did not observe significant changes in LVEDV in the SCT group. Clearly, larger studies are needed in order to confirm our results and to investigate whether these beneficial effects of SCT can be generalized to all patients with HF. Moreover, studies with a longer follow-up would be helpful in order to clarify what clinical implications of the effects produced on LA function by the training program are. In this study, which included asymptomatic or only mildly symptomatic (NYHA class I or II) HFmrEF patients, PALS and PACS were reduced compared to normal values [35], while E/e’ was still in its normal range. Considering that these patients had normal LA size and were in sinus rhythm, reservoir and contraction strains were the only detectable LA abnormality encountered in this population. These findings seem to confirm previous reports stating that LA dysfunction is present at a very early preclinical, stage in HF [13]. Our results are in agreement with the study by Saikhan et al. [36], in which all indices of LA function obtained through speckle tracking echocardiography were lower in patients with HFmrEF compared to those with preserved EF and to healthy subjects. Our preliminary data suggest that SCT elicits LA and LV functional reverse remodelling in patients with HFmrEF. In our study, the benefits of SCT on LA function appeared after 12 weeks of SCT, which can be considered a very short period of training. However, similar results have already been observed by other authors in different populations. In the study by Wright et al. [27], performed on untrained healthy men, two exercise modalities—high-intensity interval training and continuous moderate-intensity training—produced a significant increase in PALS and systolic strain rate after six training sessions. Taken together, these results suggest that the benefits of exercise training on LA function appear very early, regardless of the type of training used. In addition, the high participation rate obtained by the SCT group in the present study may have played an important role in amplifying the effects of exercise training on LA function parameters.

### 4.2. Relationship between LA Function and Exercise Tolerance

We observed significant correlations between changes in PALS and the increase in the duration of the ergometric test and distance walked at 6MWT in the SCT group. These results should be interpreted with caution, since there was no significant correlation at the baseline between strain measurements and exercise capacity. Moreover, the small sample size of the present study prevented us from investigating, through multivariate regression analysis, whether the relationship between the improvement of the LA reservoir phase and the increase in exercise capacity was independent. However, this finding deserves further investigations, since it is in agreement with recent research showing that the LA reservoir strain is an independent predictor of exercise capacity across the entire range of EF values [37,38,39]. In our study, distance walked at 6MWT increased in both SCT and CON. However, only patients performing exercise in a supervised setting obtained significant effects on the indices of LA and LV strain. Our results comply with other research, in which supervised exercise training programs were compared with unsupervised exercise prescribed in routine medical consultations [40,41]. In the study by Fabri et al. [37], patients undergoing supervised combined exercise improved LVEF at the end of the training program, while no effects on cardiac parameters were observed in the unsupervised group. Our results underline the importance of performing supervised training programs for patients with HFmrEF in order to develop adequate training intensities and to optimize the effects of exercise on cardiac function.

### 4.3. Study Strengths

This study has two main strengths. Firstly, it is the first research exploring the potential effects of a concurrent training program on LA function in patients with HFmfEF. Secondly, it helps to clarify the burden of atrial functional changes occurring in asymptomatic patients with HFmrEF.

### 4.4. Study Limitations

This study has been conceived as a pilot study; therefore, it has a small sample size, and its results should be confirmed in further, adequately powered studies. In particular, we enrolled a very small proportion of women, which should be better represented in further trials. Our results were obtained in patients undergoing concurrent exercise training, and cannot be generalized to different exercise modalities. The control group of this study included patients who performed a certain degree of physical activity, and this explains why we observed a rise in fitness in both arms; the lack of a proper control group, without exercise, may have weakened the possibility to observe the neat effect of SCT on LA function parameters. However, the problem of having a control group without physical activity is a common challenge in exercise trials, as it would be unethical not to promote physical activity given its benefits. This study enrolled only patients with HFmrEF with underlying CHD; therefore, its result cannot be extended to patients with severe LVEF or those with preserved LVEF.

## 5. Conclusions

The present study showed that SCT was superior to CON in improving functional capacity, LA and LV function in patients with HFmrEF. Further, larger studies are needed in order to confirm our results and to clarify whether these exercise-induced benefits are sustained overtime and can translate into clinical benefits for HF patients.

## Figures and Tables

**Table 1:** Baseline anthropometric and clinical characteristics of the two study groups.

	SCT (N = 35)	CON (N = 35)	*p*
Anthropometric characteristics			
Age, years	69.4 ± 10.3	70.3 ± 12.5	0.342
Males/females, n	29/6	28/7	0.223
BMI, kg/m^2^	27.6 ± 8.1	28.0 ± 6.9	0.091
Waist circumference, mm	106.8 ± 16.1	107.5 ± 14.2	0.167
Previous AMI, n (%)	28 (80)	26 (74)	0.098
Previous PCI/CABG, n	29/11	27/15	0.287
Comorbidities			
Hypertension, n (%)	31 (88)	32 (91)	0.332
Diabetes, n (%)	13 (37)	14 (40)	0.076
Hypercholesterolaemia, n (%)	29 (82)	27 (77)	0.081
Previous smoking habit, n (%)	24 (68)	22 (63)	0.165
Obstructive sleep apnea, n (%)	11 (31)	10 (28)	0.067
Treatment			
Antiplatelet therapy, n (%)	35 (100)	35 (100)	-
ACE-Is/ARBs, n (%)	32 (91)	30 (86)	0.079
Betablockers, n (%)	31 (88)	33 (94)	0.123
MRAs, n (%)	29 (83)	30 (86)	0.085
Nitrates, n (%)	10 (28)	8 (23)	0.321
Diuretics n (%)	12 (34)	13(37)	0.184
Statins, n (%)	32 (91)	35 (100)	0.065

PCI = percutaneous coronary intervention; CABG = coronary artery bypass grafting; ACE-I = angiotensin-converting enzyme inhibitors; ARBs = angiotensin receptor blockers. MRAs = mineralocorticoid receptor antagonists.

**Table 2:** Changes on LA and LV parameters between the two study groups.

	SCT (34)		CON (31)	
	Baseline	12 Weeks	Baseline	12 Weeks
PALS, %	21.4 ± 4.3	27.6 ± 5.1 *°	21.8 ± 5.6	22.7 ± 6.0
Conduit, %	14.6 ± 4.3	19.6 ± 5.2 *°	15.2.6 ± 4.7	14.9 ± 3.8
PACS, %	16.8 ± 7.3	20.4 ± 8.1 *°	16.3.6 ± 6.4	18.7 ± 6.9
LAVI, mLm^2^	26.2 ± 4.5	25.8 ± 5.8	26.0 ± 6.8	26.3 ± 5.0
E wave, cm/s	57.7 ± 17.8	58.1 ± 14.4	57.3 ± 15.8	58.7 ± 19.4
A wave, cm/s	59.3 ± 20.0	60.2 ± 22.8	61.4 ± 17.4	60.2 ± 22.3
E/A ratio	0.97 ± 0.5	0.96 ± 0.8	0.93 ± 0.7	0.97 ± 0.3
LVEDV, mL	124.2 ± 39.5	127.1 ±39.5	125.7 ±39.5	124.1 ± 39.5
LVESV, mL	69.5 ± 26	65.8± 22.6	67.1 ± 26	66.3± 22.6
E/e’ ratio	8.3 ± 1.8	7.8 ± 1.5	8.8 ± 2.2	8.4 ± 1.9
LVEF, %	45.2 ± 3.9	46.9 ± 4.7	46.2 ± 6.3	46.6.6 ± 5.8
GLS, %	−16.5 ± 6.3	−21.4 ± 7.5 *°	−16.1 ± 5.8	−17.5 ± 8.7
SV, mL	56.5 ± 17.0	58.2 ± 15.1	58.4 ± 17.3	57.8 ± 14.6
HR, bpm	65.4 ± 12.2	63.5 ± 16.7	64.6 ± 14.6	65.0 ± 15.9
CO, l/min	3.6 ± 1.3	3.7 ± 1.8	3.7 ± 1.5	3.7 ± 2.1

PALS = peak atrial longitudinal strain; PACS = peak atrial contraction strain; LAVI = left atrial volume index; LVEF = left ventricle ejection fraction GLS = global longitudinal strain. SV = stroke volume; HR = heart rate; CO = cardiac output. * Intragroup *p* < 0.05; ° between-group *p* < 0.05.

## Data Availability

The data presented in this study are available on request from the corresponding author.
